# Uncoupled nitric oxide synthase activity promotes colorectal cancer progression

**DOI:** 10.3389/fonc.2023.1165326

**Published:** 2023-03-14

**Authors:** Asim Alam, Steven C. Smith, Sundaresan Gobalakrishnan, Mina McGinn, Vasily A. Yakovlev, Christopher S. Rabender

**Affiliations:** ^1^ Department of Radiation Oncology, Virginia Commonwealth University, Richmond, VA, United States; ^2^ Department of Pathology, Virginia Commonwealth University, Richmond, VA, United States; ^3^ Department of Radiology, Virginia Commonwealth University, Richmond, VA, United States

**Keywords:** colorectal cancer, nitric oxide synthase, reactive oxygen species, reactive nitrogen species, tetrahydrobiopterin

## Abstract

Increased levels of reactive oxygen/nitrogen species are one hallmark of chronic inflammation contributing to the activation of pro-inflammatory/proliferative pathways. In the cancers analyzed, the tetrahydrobiopterin:dihydrobiopterin ratio is lower than that of the corresponding normal tissue, leading to an uncoupled nitric oxide synthase activity and increased generation of reactive oxygen/nitrogen species. Previously, we demonstrated that prophylactic treatment with sepiapterin, a salvage pathway precursor of tetrahydrobiopterin, prevents dextran sodium sulfate–induced colitis in mice and associated azoxymethane-induced colorectal cancer. Herein, we report that increasing the tetrahydrobiopterin:dihydrobiopterin ratio and recoupling nitric oxide synthase with sepiapterin in the colon cancer cell lines, HCT116 and HT29, inhibit their proliferation and enhance cell death, in part, by Akt/GSK-3β–mediated downregulation of β-catenin. Therapeutic oral gavage with sepiapterin of mice bearing azoxymethane/dextran sodium sulfate–induced colorectal cancer decreased metabolic uptake of [^18^F]-fluorodeoxyglucose and enhanced apoptosis nine-fold in these tumors. Immunohistochemical analysis of both mouse and human tissues indicated downregulated expression of key enzymes in tetrahydrobiopterin biosynthesis in the colorectal cancer tumors. Human stage 1 colon tumors exhibited a significant decrease in the expression of quinoid dihydropteridine reductase, a key enzyme involved in recycling tetrahydrobiopterin suggesting a potential mechanism for the reduced tetrahydrobiopterin:dihydrobiopterin ratio in these tumors. In summary, sepiapterin treatment of colorectal cancer cells increases the tetrahydrobiopterin:dihydrobiopterin ratio, recouples nitric oxide synthase, and reduces tumor growth. We conclude that nitric oxide synthase coupling may provide a useful therapeutic target for treating patients with colorectal cancer.

## Introduction

Wnt signaling is a critical regulator of normal cell turnover in healthy colonic epithelial cells. Thus, it is not surprising that more than 90% of spontaneous colorectal cancer (CRC) cases have activating mutations in Wnt signaling in the early stages of tumor progression (1). The major effector of Wnt signaling in both normal and malignant colonic epithelial cells is β-catenin, and the most common activating mutation of Wnt signaling in CRC is in the adenomatous polyposis coli (APC) gene. In the absence of Wnt ligand, APC binds to phosphorylated β-catenin, forming a destruction complex in which β-catenin is degraded by the proteasome (2). There are other activating mutations of the Wnt pathway including constitutive activation of Wnt receptor without ligand, mutated forms of β-catenin unable to bind the APC protein, and loss of function of glycogen synthase kinase-3β (GSK-3β), the kinase responsible for β-catenin phosphorylation (3). Regardless of the underlying mechanism, an important consequence of aberrant Wnt signaling is nuclear accumulation of β-catenin. Cooperating with members of the TCF/LEF transcription factor family, β-catenin activates a number of target genes including Cyclin D1 (CCND1), cell division cycle 25a (CDC25A), Claudin-7 (CLDN7), vascular endothelial growth factor (VEGF), and matrix metalloproteinase 7 (MMP7), that modulate cell proliferation, metastasis, and tumor progression (1).

Patients with inflammatory bowel diseases have a 5- to 15-fold increased risk of developing CRC in their lifetimes (4). The tumors arising in these patients are difficult to treat and have a >50% mortality rate (5, 6). Patients with inflammatory bowel disease and a family history of CRC are also twice as likely to develop cancer compared with patients with inflammatory bowel disease without a positive family history (7). The histopathology of colitis-associated CRC is similar to hereditary or sporadic CRC, and similar genetic mutations in the Wnt signaling pathway leading to increased nuclear accumulation of β-catenin are also present in colitis-associated CRC. Furthermore, there are robust pro-inflammatory infiltrates and increased pro-inflammatory cytokine levels in CRC without any clinically detectable signs of gastrointestinal distress (3). These findings suggest a mechanistic overlap and a dependence on an inflammatory microenvironment in all etiologies of CRC.

A hallmark of an inflammatory microenvironment is increased levels of reactive oxygen/nitrogen species (ROS/RNS). The major sources of ROS/RNS are the three isoforms of nitric oxide synthase (NOS), neuronal (nNOS), inducible (iNOS), and endothelial (eNOS). These isoforms are differentially expressed depending on tumor type, but all three have common cofactor requirements including tetrahydrobiopterin (BH4), nicotinamide adenine dinucleotide phosphate (NADPH), Flavin adenine dinucleotide (FAD), and flavin mononucleotide (FMN) (8, 9). Under normal physiological conditions with a complete complement of cofactors and substrate, NOS produces nitric oxide (•NO). At the •NO concentrations produced by nNOS and eNOS, •NO binds to the heme of soluble guanylate cyclase initiating Guanosine 3’,5’-cyclic monophosphate (cGMP) synthesis from Guanosine 5’-triphosphate (GTP) and activation of protein kinase G. By this mechanism, •NO acts as principle vasoregulator stimulating phosphorylation of the vasodilator-stimulated phosphoprotein and inositol 1,4,5-triphosphate receptor (10). •NO/protein kinase G signaling is also involved in cell proliferation inhibiting both non-canonical transforming growth factor–β signaling and the Wnt/β-catenin pathways. Thus, sildenafil and other inhibitors of phosphodiesterases that hydrolyze cGMP suppress inflammation-driven CRC progression (11–14).

Although well described in the vasculature literature, studies of NOS in cancer cells generally ignore the fact that NOS can have two activities: “coupled” that generates •NO or “uncoupled” that generates ${\text{O}}_{2}^{-}/{\text{ONOO}}^{-}$ . A key factor determining the state of coupling is the ratio of [BH4] to its oxidation product [BH2] because both bind to NOS with equal affinity. When the BH4:BH2 ratio is low as found in inflammatory conditions, uncoupling is observed and more ${\text{O}}_{2}^{-}/{\text{ONOO}}^{-}$ and less •NO are generated (15, 16). Because ONOO^−^ oxidizes BH4 to BH2, a futile feed forward destruction mechanism of BH4 can be established. This switch in activities can have important consequences for downstream pro- and anti-growth signaling pathways.

We (17) recently showed that diverse tumor cell types *in vitro* and *in vivo* have low BH4:BH2 ratios (≤2) compared to normal tissues, including human colorectal tumor biopsies compared with paired adjacent “normal tissue” biopsies (>4). Furthermore, when sepiapterin (SP), a BH4 salvage pathway precursor, was included in the medium of cultured cells or provided orally to animals bearing tumors cells, the BH4:BH2 ratio increased in tissue culture, in breast cancer xenografts, and in a spontaneous MMTVneu breast tumor model. SP enhanced tumor cell killing both *in vitro* and *in vivo* in these breast cancer models. Previously, we also demonstrated that administering SP prophylactically in the azoxymethane dextran sodium sulfate (AOM/DSS) mouse model for colitis-associated CRC inhibited not only protein tyrosine nitration as a marker of NOS uncoupling but also reduced both colitis and tumor development (18). Herein, the therapeutic administration of SP is shown to inhibit CRC growth through a mechanism targeting Akt-β-catenin signaling. Furthermore, the observed changes in the expression levels of key biopterin metabolic enzymes provide a mechanism for sustaining a low BH4:BH2 ratio during CRC progression in mice and in humans.

## Materials and methods

### Reagents and tissue culture

L-Sepiapterin was purchased from Schirks, Laboratories (Jona, Switzerland), Nω-nitro-L arginine, Euk134 and S-nitrosoglutathione from Sigma-Aldrich (St Louis, MO), and GP91ds-tat from Anaspec (Fremont, CA). The following primary antibodies (and sources) were used: goat anti-actin (sc-1615, Santa Cruz Biotechnology, Dallas, TX) and mouse monoclonal anti-GAPDH (MAB374, Millipore, Burlington, MA); Cell Signaling Technology (Danvers, MA) provided rabbit polyclonal anti-cdc25A (cst-3652), rabbit polyclonal anti-Akt (cst-9272), rabbit polyclonal anti pS^33/37^-β-catenin (cst-2009), rabbit monoclonal anti-non-phospho (active) β-catenin (cst-8814), mouse monoclonal anti-pS^9^-GSK3β (cst-9832), and rabbit monoclonal anti-GSK3β (cst-9323) and anti-pS^473^-Akt (cst-4060). Other antibodies included mouse monoclonal anti-β-catenin (#610153, BD Transduction Laboratories, San Jose, CA); anti-CBR1, anti-CBR3, and anti-AKR1C3 rabbit polyclonal antibodies (A5446, A7545, and A13568, ABclonal, Woburn, MA); rabbit polyclonal anti-SPR, and anti-quinoid dihydropteridine reductase (QDPR) (#22293 and 28041, ThermoFisher Scientific, Waltham, MA) and mouse monoclonal anti-dihydrofolate reductase (DHFR) (#MAB7934, R&D Systems, Minneapolis, MN). The Apotag kit was from Millipore. CRC cell lines (HCT116 and HT29) were purchased from American type culture collection (ATCC) and grown as monolayers in McCoy’s 5A medium (Thermo Fisher) supplemented with 10% fetal bovine serum (FBS) and penicillin and streptomycin (50 U/ml). Experiments were performed only on cells grown up to 10 passages. Clonogenic assays were performed as described (17).

### Induction of colitis and CRC

Colitis/carcinogenesis was induced by the intraperitoneal injection of AOM (10 mg/kg) followed by three cycles of 2% dextran sodium sulfate (DSS) treatment as previously described (18). Two weeks after the last DSS treatment, animals were treated with three daily doses of SP (every 8 h) for a total daily dose of 1 mg/kg/day *via* oral gavage for 8 days. A subset of animals were treated with SP in their drinking water (170 μM SP) to provide an approximate dose of 0.64 mg/kg/day for 3 weeks as previously described (18) All procedures were approved by the Institutional Animal Care and Use Committee of Virginia Commonwealth University (protocol number AM10185) and conformed to the guidelines established by the National Institutes of Health.

### Biochemical analyses

For all analyses involving SP [clonogenic assays, Western blot, and high performance liquid chromatography (HPLC) experiments] a stock solution of fresh 1 mM SP with 0.25% dimethyl sulfoxide (DMSO) in a serum-free culture medium was made and subsequently diluted appropriately. For control/untreated experimental conditions, a serum-free culture medium with 0.25% DMSO was diluted appropriately depending on amount used from stock 1 mM SP solution. Fresh SP and media were replaced daily for long course treatment with SP.

Biopterin measurements of cultured cells were as described using HPLC analysis (17). To analyze tumor biopterin levels *in vivo*, mice were anesthetized and euthanized by cervical dislocation. The distal colon was excised, and colonic epithelial cells were separated from the mucosal layer and tumor polyps as described (19). Tumors were surgically excised after removal of epithelial cells. The tumors or normal epithelial cells were either placed in the −80°C freezer or immediately homogenized in 10 volumes of 0.1N HCl with a pestle and mortar on ice. The homogenates were centrifuged for 20 min at 13,200 rpm, and the supernatants were stored at −80°C prior to HPLC analysis.

GSK3β shRNA Lentiviral Particles-A and control shRNA Lentiviral Particles-A were purchased from Santa Cruz Biotechnology (Dallas, TX). In a 12-well plate, HCT-116 cells were plated in complete McCoy’s medium with 10% FBS to achieve ~50% confluence the following day. Complete medium (1 ml per well) with Polybrene (5 µg/ml; sc-134220) and appropriate lentiviral particles (20 µl) were prepared as infection medium. Complete growth medium was replaced with infection medium and incubated overnight for 12 h. The medium was changed to a fresh complete growth medium and incubated overnight. To select stable clones, cells were split 1:3 from original 12-well plates and grown for 48 h in 10-cm dishes. shRNA expressing clones were selected by treating dishes with puromycin dihydrochloride (10 µg/ml) in a complete growth medium. Fresh puromycin was replaced every 3–4 days. After 2 weeks of growth under puromycin selection, cells were split 1:50 into multiple dishes. Single colonies were picked under sterile conditions and grown to confluence under puromycin selection. These clones were then analyzed *via* Western blot analysis for GSK3β expression.

TCF/LEF promoter activity was measured with a luciferase reporter assay using HCT-116 cells as described by the manufacturer (Addgene, Cambridge, MA). Briefly, for T cell factor/lef-1 family of transcription factors (TCF/LEF) promoter activity, cells were treated with SP. Twenty-four hours before analysis cells were transfected by lipofectamine/plus with a luciferase tagged TCF/LEF reporter construct (Addgene) in SP-free medium. Three hours later, the medium was changed with fresh SP and luciferase activity measured 24 h later.

S-nitrosylated proteins were purified and analyzed as previously described using biotin switch methodology (20).

### Immunochemical analysis

For immunohistochemical analysis, mouse colons were prepared as “Swiss rolls” (18). In some experiments, tumors were excised from the colons, rinsed in phosphate buffered saline (PBS), and frozen in liquid nitrogen. Swiss rolls or excised tumors were embedded in optimal cutting temperature (OCT) medium and cryosections prepared. De-identified human patient colon samples frozen in OCT were obtained from the Massey Cancer Center Tissue and Data Acquisition and Analysis Core through a local Institutional review board (IRB)-approved protocol that abides by the Declaration of Helsinki principles. Multiple frozen sections were fixed in ice-cold 4% paraformaldehyde in PBS for 10 min. The middle section was used for Hematoxylin & Eosin (H&E) staining and delineation of normal and cancer areas. Sections on either side were stained for the different proteins as follows. Mouse sections were blocked with the M.O.M. kit from Vector Laboratories (Burlington, CA). Human samples were blocked with goat serum for 60 min. Sections were stained with primary antibody at 1:200 dilution overnight at 4°C, followed by incubation with Alexa-conjugated appropriate secondary antibody at 1:500 dilution at room temperature for 1 h. After washing, sections were stained with 4′,6-diamidino-2-phenylindole (DAPI). Images were captured using the Ariol Digital Pathology Platform. Quantitation was achieved with ImageJ software using nuclear DAPI staining for normalization.

### PET/CT imaging

For positron emission tomography/computed tomography (PET/CT) imaging animals were fasted, anesthetized (2% isoflurane in oxygen), and injected intravenous (iv) with 300 µCi [^18^F]-FDG (fluorodeoxyglucose) from IBA Molecular (Sterling, VA). After 60 min of FDG uptake, animals were positioned in the Inveon Preclinical System (Siemens Healthcare, Malvern, PA), and PET/CT images were acquired for 10 min with no attenuation correction. The PET images were processed using manufacturer recommended calibration procedures and a phantom of known volume and activity acquired prior to the study. OSEM3D-MAP reconstructions were done using Inveon Acquisition Workplace 1.5 and were used for region-of-interest analysis in the Inveon Research Workplace 4.1. The percent injected dose/gram of tissue (%ID/g) values were calculated after appropriate decay corrections using the formula, %ID/g = C_t_/ID × 100, where C_t_ is the concentration of radiotracer in the tissue (MBq/cc), obtained from the PET mages after region-of-interest analysis.

## Results

### SP increases the BH4:BH2 ratio in CRC cells and decreases proliferation *in vitro* and *in vivo*

**Figure 1:**
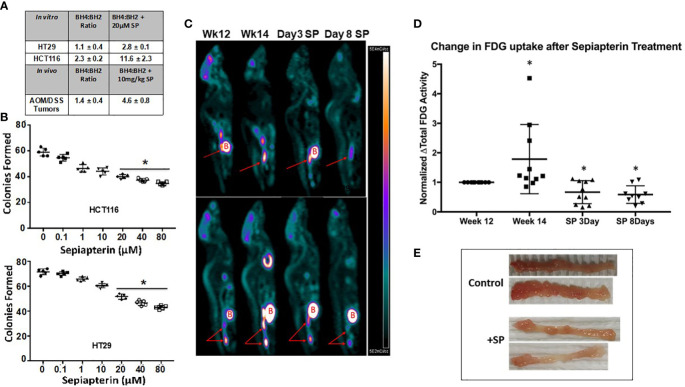
SP raises the BH4:BH2 ratio in CRC cells and decreases proliferation both *in vitro* and *in vivo.* **(A)** BH4:BH2 ratios were measured *in vitro* cell lines and from tumors obtained from AOM/DSS mice treated with and without SP at 1 mg/kg. Values are given ± SEM. **(B)** Cells were treated with SP for 24 h, plated and grown to viable colonies of 50 or more cells. Statistical significance was evaluated with the Student’s t-test. *p<0.05. **(C)** At 2 weeks following completion of three cycles of DSS treatment, animals were imaged to obtain baseline tumor FDG activity. Two weeks later, animals were re-imaged to assess tumor progression by [^18^F]-FDG tumor activity. Animals were then treated with SP by oral gavage (1 mg/kg/day) and imaged at 3 and 8 days. (Red arrows: tumor; B = Bladder) **(D)** [^18^F]-FDG uptake values were calculated and normalized to the fold change from week 12 and averaged for N = 10 animals. Fold change values are shown as ± SD. *p ≤ 0.05 using Student’s t-test. **(E)** At 2 weeks after final DSS treatment, animals were administered with SP (4 mg/L) in their drinking water for 3 weeks after which the animals were sacrificed and colons collected.

Our previous studies demonstrated in HT29 human colon cancer cells *in vitro* and in paired tumor and adjacent normal human colon biopsies that the BH4:BH2 ratio is lower in the tumor cells relative to normal tissue (17). To extend these findings, the BH4:BH2 ratio was measured in another colon cancer cell line, HCT116, and in isolated tumors from the colons of AOM/DSS-treated mice. **Figure 1A** shows that incubating HCT-116 cells with 20 µM SP for 24 h significantly increased the BH4:BH2 ratio, 2.3 ± 0.2 to 11.6 ± 2.3. SP treatment elevated the BH4:BH2 ratio by more than 2.5-fold in HT29 cells and about five-fold in HCT-116 cells compared to baseline.

A colony formation assay was used to determine whether SP has effects on the proliferation capacity of tumor cells *in vitro*. Increasing concentrations of SP resulted in a progressive reduction in colony formation in both HCT-116 and HT29 cell lines (**Figure 1B**). At the SP concentration used in subsequent experiments, 20 μM, there is a 30%–40% decrease in colonies compared to untreated controls.

*In vivo*, after the final round of DSS treatment, animals were returned to normal drinking water for 4 weeks to allow for colitis to subside and tumors to continue growing. Animals were then treated with SP for 8 days after which the tumors were excised and the BH4:BH2 ratio determined by HPLC. In all cases the BH4:BH2 ratio of tumor cells was significantly lower than that of normal mouse colon epithelial cells (7.1+/−0.6 [19], **Supplementary Information Table 1**). *In vivo*, there was an approximate three-fold increase in the BH4:BH2 ratio in the tumors from SP-treated animals (**Figure 1A**).

[^18^F]-FDG/PET/CT imaging was used to determine the effects SP on CRC progression *in vivo*. Two weeks after the last DSS treatment and prior to SP treatment, animals were imaged two times separated by 14 days (N = 10). Animals were then imaged on days 3 and 8 of SP treatment (1 mg/kg/day by oral gavage). **Figures 1C, D** show representative PET images at different time points and the quantification of PET images from 10 animals normalized to their initial [^18^F]-FDG uptake at week 12, respectively. There is an approximate two-fold increase in [^18^F]-FDG uptake during the 2 weeks without any treatment followed by a 40% reduction in [^18^F]-FDG uptake from baseline after 3 and 8 days of SP treatment.

The effect of SP on tumor burden was also assessed by gross examination and by assay for apoptosis in tumor-bearing animals treated with SP for a longer duration. Two weeks after the last DSS administration, the drinking water of half the mice was supplemented with 170 μM SP for 3 weeks at which time the mice were sacrificed. **Figure 1E** shows with two representative colons that SP for 3 weeks reduced the number of tumors per colon. In agreement, **Supplementary Information Figure 1** shows an increase in tumor apoptosis measured by immunofluorescence. Collectively, these data suggest that SP has anti-proliferative and cytotoxic effects on CRC cell lines *in vitro* and spontaneous colitis-associated cancer *in vivo*.

### SP treatment decreases expression of β-catenin protein both *in vitro* and *in vivo*

**Figure 2:**
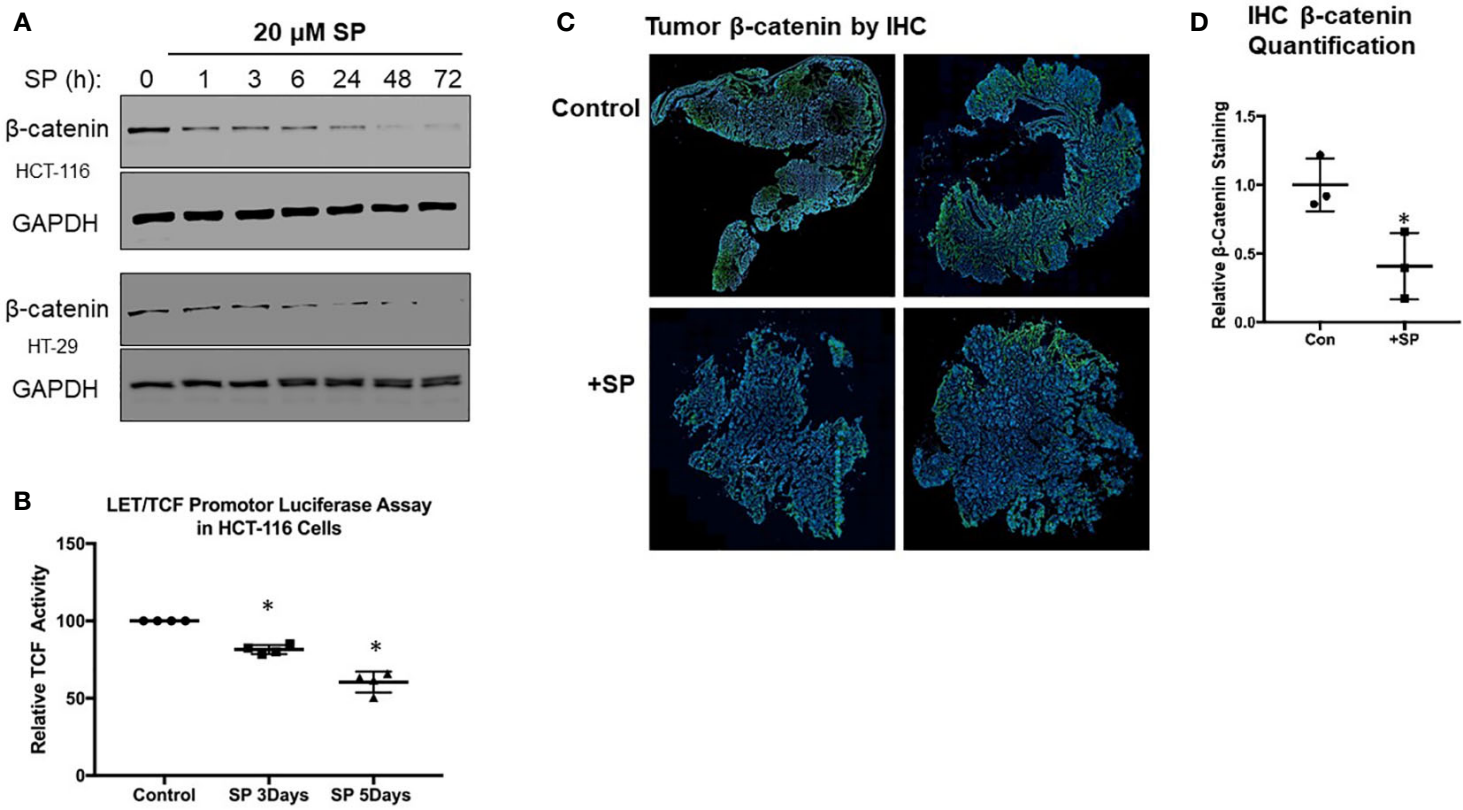
SP decreases β-catenin expression *in vivo and ex vivo.* **(A)** Western blot analysis for active (non-phosphorylated) β-catenin expression in HTC-116 and HT-29 cells treated with 20 µM SP. **(B)** LET/TCF luciferase promotor activity assay in HCT-116 cells incubated with 20 µM SP for 3 and 5 days. Results are normalized to untreated cells and reported as ± SD. Student’s t-test was used to assess statistical significance (*p< 0.05). **(C)** *Ex vivo* immunofluorescence analysis of β-catenin in CRC tumors. At 2 weeks after the final DSS treatment, animals were administered SP in their drinking water for 3 weeks as described in Materials and Methods. Tumors were excised from colon, and histological slides were made and stained for β-catenin expression. Representative sections are shown with β-catenin staining in green and DAPI nuclear staining in blue. **(D)** Quantification of β-catenin fluorescence was normalized to DAPI for control (N = 3) and SP treated (N = 3). Values are shown as ± SD; *p< 0.05 by t-test.

The Wnt signaling pathway involving β-catenin transcriptional activity is a key driver of epithelial tumor progression. Our previous studies demonstrated that SP downregulated β-catenin protein expression in breast cancer cells (17). We tested whether SP also reduced β-catenin protein levels in HT29 and HCT-116 cell lines. As shown in **Figure 2A**, the level of active (non-phosphorylated) form of β-catenin protein in both HT29 and HCT116 cells decreased within 1 day of SP treatment. To confirm that SP was able to decrease the transcriptional activity of β-catenin, a LEF/TCF luciferase promoter activity assay was employed. In HCT-116 cells, LEF/TCF-driven luciferase expression was significantly inhibited after 3 days of SP treatment (**Figure 2B**), with similar results in HT29 cells (data not shown).

We obtained similar results in the AOM/DSS-treated animals with respect to β-catenin expression. Immunofluorescence staining (**Figures 2C, D**) shows that tumor β-catenin protein expression is significantly downregulated after 3 weeks of consuming drinking water supplemented with 170 μM SP. In summary, both *in vitro* and *in vivo* experiments demonstrate that SP treatment results in reduced β-catenin protein levels.

### SP treatment leads to S-nitrosylation of Akt and decreased Akt activity

**Figure 3:**
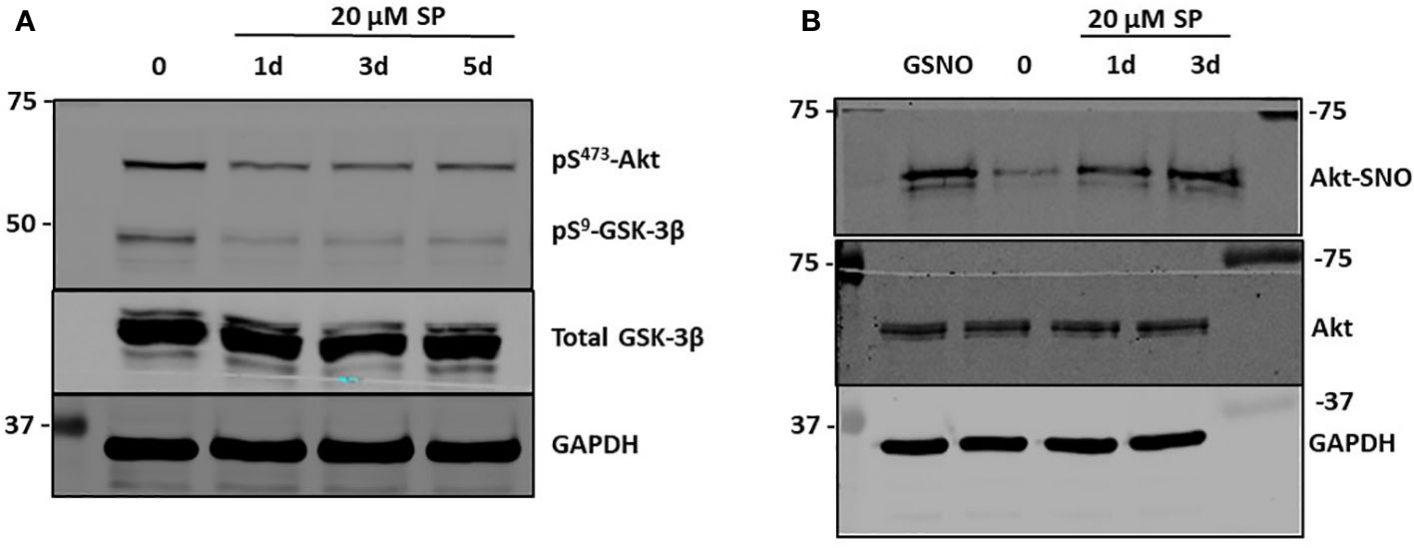
SP treatment leads to S-nitrosylation of AKT and decreased pSer^473^ AKT. **(A)** Western blot analysis of pAkt and pGSK3β after 20 µM SP treatment in HCT-116 cells. **(B)** The biotin switch assay, previously described (20), was used to determine s-nitrosylation of Akt in HCT-116 cells with 20 µM SP treatment after 1 or 3 days. As a positive control s-nitrosoglutathione (GSNO), a nitric oxide (NO) donor, was added to the cell lysates for 5 min. Representative blots from two separate experiments are shown.

β-catenin is targeted for proteomic degradation after phosphorylation by GSK3β. Akt is a potent activator of Wnt signaling by phosphorylating and inhibiting GSK3β, thereby preventing proteasomal β-catenin degradation (21). Because Akt is activated during times of oxidative stress, and SP decreases oxidative stress by increasing the BH4:BH2 ratio and *NO synthesis while reducing generation of oxidants ${\text{O}}_{2}^{-}$ and ONOO^−^, we tested whether Akt activity was inhibited by SP. Phosphorylation of Akt at Ser^473^ (a marker for its activation) was assayed by Western blot analysis in HCT-116 and HT-29 cells. Phosphorylation at Ser^473^ was decreased with SP treatment in both cell lines (**Figure 3A**; **Supplemental Information Figure 2A**). Given the published reports that show •NO donors inactivate Akt by S-nitrosylation of Cys^224^, we tested whether incubating HCT116 cells with either the *NO donor, S-nitrosoglutathione, or with SP stimulated S-nitrosylation of Akt and thereby inhibited its activity (22). As shown in **Figure 3B**, treating cells with either a *NO donor or SP results in S-nitrosylation of Akt. The loss of Akt activity was confirmed by assaying for GSK3β Ser^9^ phosphorylation, a known Akt phosphorylation site (23). For both HCT-116 and HT-29 cells, incubation with SP blocked phosphorylation of Ser^9^-GSK3β (**Figure 3A**; **Supplementary Information Figure 2A**). To confirm increased activity of GSK3β, Ser^33/37^ phosphorylation of β-catenin and expression of cdc25A, which is also degraded as a consequence of GSK3β activity (24), were analyzed by Western blot (**Supplementary Information Figure 2B**). Phosphorylation of β-catenin was enhanced in SP- treated HCT-116 cells by a mechanism sensitive to the GSK3β inhibitor, SB216763. Expression levels of cdc25A were also decreased in cells incubated with SP. Thus, in these CRC cells, SP leads to decreased activation of AKT and a corresponding increased activation of GSK3β contributing to β-catenin degradation.

### Inhibiting GSK3β expression blocks SP effects on β-catenin, cdc25A expression, and HCT116 growth

**Figure 4:**
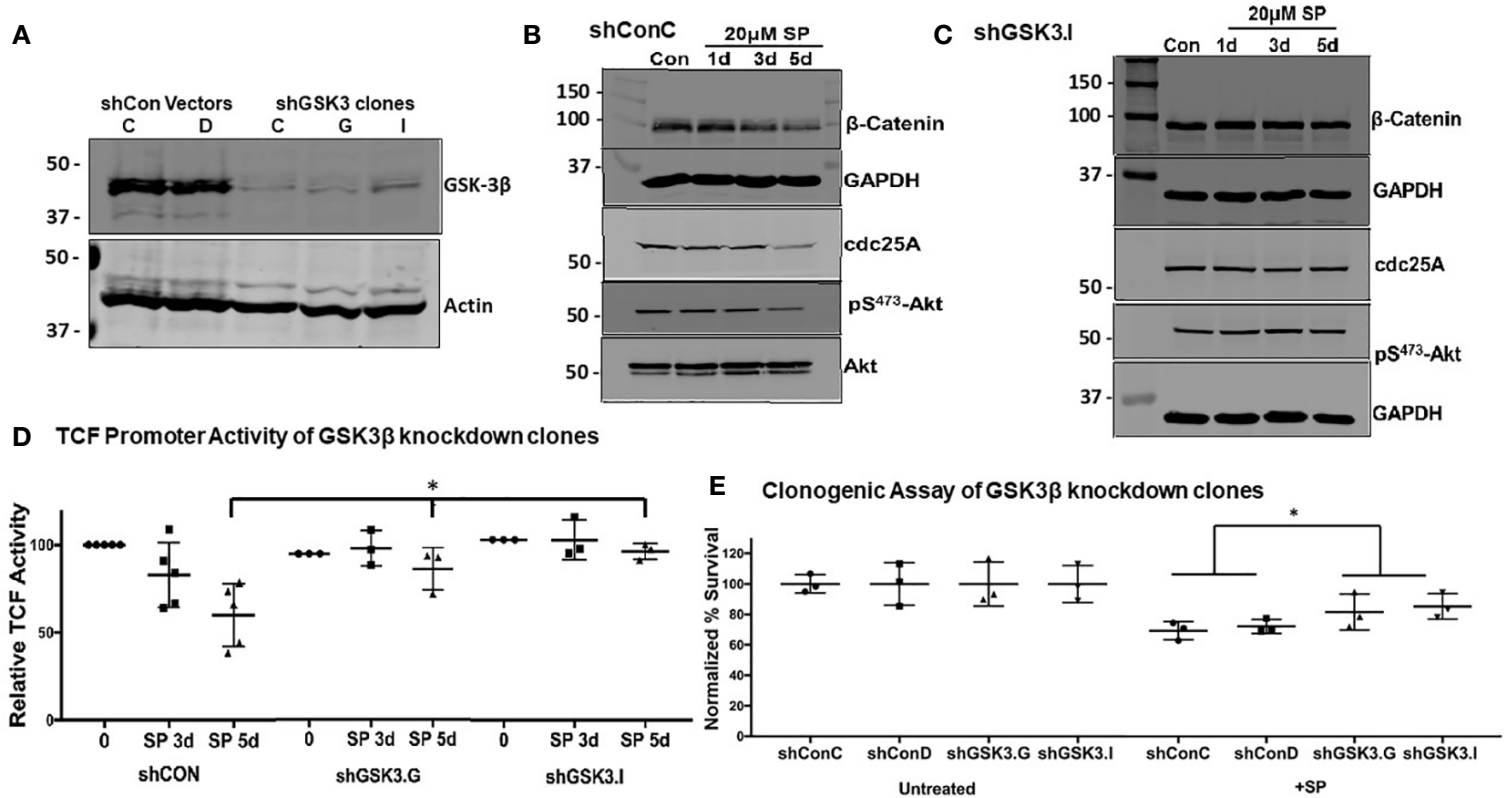
GSK-3β knockdown mitigates the effects of SP in HCT-116 cells: **(A)** Confirmation by Western blot of GSK-3β knockdown in shGSK3.I and shGSK3.G clones. shConC **(B)** cells or shGSK3.I **(C)** cells were treated with 20 µM of SP for 1, 3, or 5 days, and protein expression of β-catenin, cdc25A, and pSer^473^-Akt were measured by Western blot. Representative blots are shown from three separate experiments. **(D)** LEF-TCF promotor activity was measured in shConC, shGSK3.G, and shGSK3.I clones. Results are normalized to untreated cells and reported as ± SD. Student’s t-test was used to assess for statistical significance; *p<0.05. **(E)** For *in vitro* clonogenic assays, scrambled and GSK3β knockdown HCT-116 cells were treated with 20 µM SP for 24 h before plating and growing colonies to 50 cells or more. Colony counts were normalized to untreated cells and assessed for statistical significance using the Student’s t-test. *p< 0.05.

To confirm GSK3β’s role in SP’s anti-proliferative effects, HCT-116 cells were stably transfected with shRNA to knockdown GSK3β expression, with several clones isolated (**Figure 4A**). Subsequent experiments compared the effects of SP on cells expressing scrambled shRNA (clones shConC or shConD) and GSK3β (shGSK3.I or shGSK3.G) knockdown cells. With genetic knockdown of GSK3β, the reduced expression of β-catenin and cdc25A observed with SP treatment is lost [compare **Figure 4B** (shconC) and **Figure 4C** (shGSK3.I)]. Similarly, GSK3β knockdown prevented SP inhibition of LEF/TCF luciferase activity without effecting overall LEF/TCF activity (**Figure 4D**). We also tested the effects of GSK3β knockdown on the cytotoxic activity of SP. A colony-forming assay shows that the cytotoxic activity of SP is partially suppressed by inhibiting GSK3β expression (**Figure 4E**). This suggests that the cell growth inhibiting activity of SP involves more than the Akt/GSK3β pathway.

### An oxidative environment and biopterin metabolism contribute to a decreased BH4:BH2

After a myocardial infarction, eNOS becomes uncoupled *via* the oxidation of BH4 to BH2 in cardiac tissue. Several groups have postulated that this oxidation is due to an increase in ${\text{O}}_{2}^{-}$ and ONOO^−^ resulting from ischemia and inflammatory cell infiltration (25). Given the inflammatory microenvironment and hypoxic nature of tumors, we tested whether ROS/RNS have a role in decreasing the BH4:BH2 ratio. For this, we used inhibitors and scavengers of ROS/RNS. Nω-nitro-l-arginine (L-NNA) is a specific inhibitor of all NOS isoforms, GP91ds-tat is a small peptide inhibitor of NADPH oxidases, which generate ${\text{O}}_{2}^{-}$ , and EUK134 is a scavenger of ${\text{O}}_{2}^{-}$ and ONOO^−^. **Supplementary Table 1** shows that these inhibitors individually or combined and at concentrations known to be effective *in vitro (* 26–28) have only minimal effects on BH4:BH2 in HCT116 cells. This suggests that there must be a fundamental change in biopterin metabolism to account for the low BH4:BH2 in these cells.

To determine a possible mechanism for the low BH4:BH2, protein expression levels of key enzymes in the *de novo* synthesis and salvage pathways of BH4 of normal colonic epithelial cells from non-tumor bearing animals and CRC cells from animals treated with AOM/DSS were compared. The four enzymes studied were GTP cyclohydrolase-1 (GCH-1), SP reductase (SPR), quinoid dihydropteridine reductase (QDPR), and dihydrofolate reductase (DHFR). GCH-1 is the enzyme responsible for catalyzing the rate limiting step in *de novo* synthesis. We previously demonstrated that overexpressing GCH-1 increased the BH4:BH2 ratio in MCF-7 breast cancer cells (17). SPR is an enzyme responsible for reducing 6-pyruvoyltetrahydrobiopterin to BH4 in the *de novo* pathway and also converting BH2 (and SP) back to BH4 in the salvage pathway. QDPR is important in both the salvage and *de novo* pathways, and, much like SPR, it is responsible for reducing upstream pteridine derivatives into BH4 (25, 29, 30). DHFR reduces BH2 to BH4 (25).

**Figure 5:**
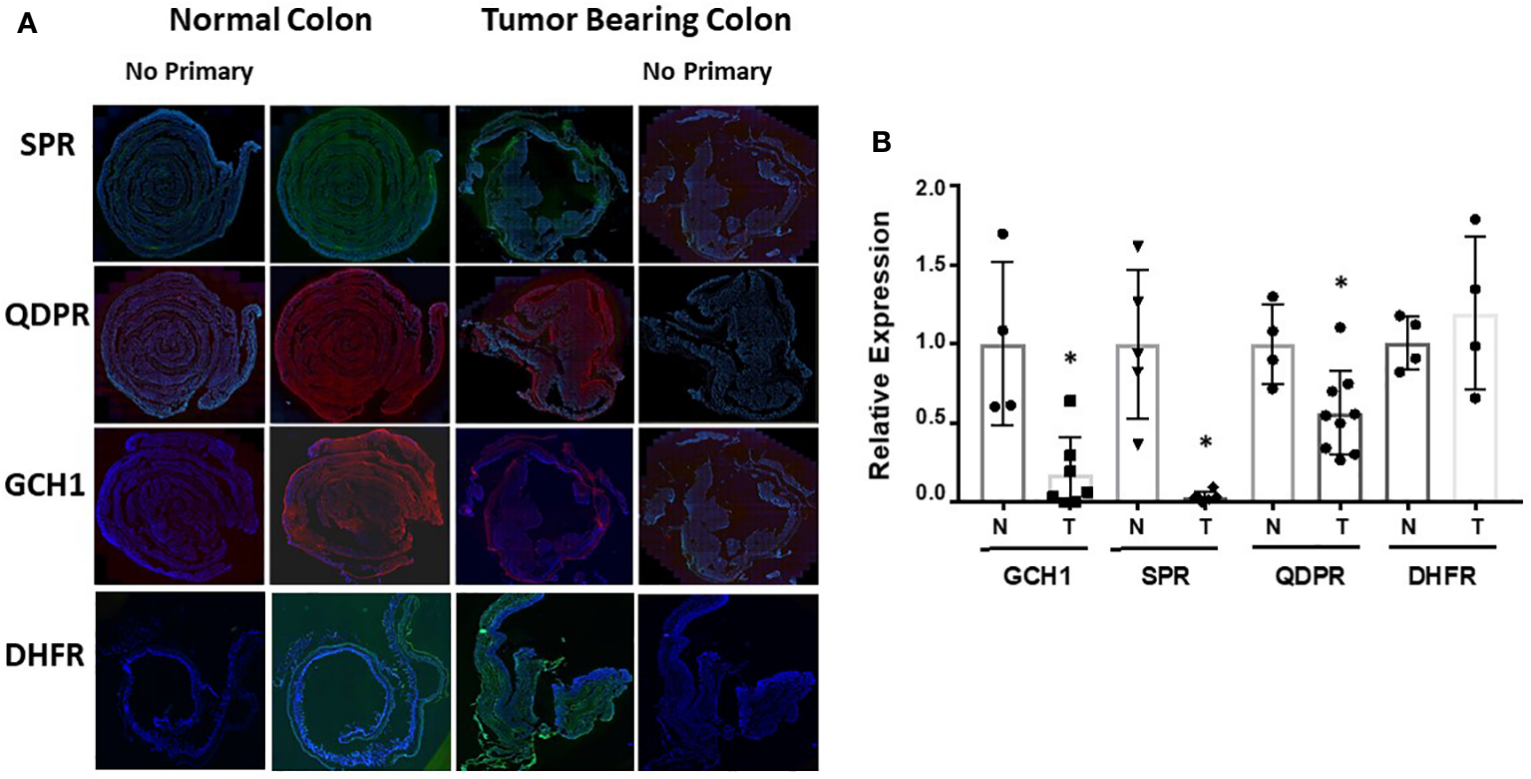
*Ex vivo* analysis of biopterin metabolic enzymes. **(A)** Colons from control or tumor bearing animals after treatment were harvested and probed for SPR, QDPR, GCH-1, and DHFR. Representative images are shown. **(B)** Quantification was done by fluorescence of specific protein normalized to DAPI fluorescence. Relative normalized expression is shown as ± SD. Student’s t-test was used to assess for statistical significance (*p<0.05).

Representative immunofluorescence staining of SPR, QDPR, GCH-1, and DHFR is shown in **Figure 5A** with quantification of staining after normalization to DAPI nuclear staining provided in **Figure 5B**. With the exception of DHFR, the normal colon expressed significantly higher amounts of these enzymes compared to the colons of tumor bearing mice. DHFR levels of normal and tumor tissues were approximately identical. These data collectively indicate that changes in expression of the BH4 synthesis and salvage pathway enzymes may be the major mechanism of how AOM/DSS induced CRC cells maintain a decreased BH4:BH2.

The decreased expression of SPR found in the mouse CRC raises questions of how exogenous SP is converted to BH2 and ultimately to BH4 through further reduction. Previous studies have demonstrated that aldose and carbonyl reductases can reduce SP to BH2 (31). To confirm that these reductases are present in AOM/DSS induced CRC, Swiss rolls and tumors were stained for CBR1, CBR3, and AKR1C3. As shown in **Supplementary Figure 4**, these enzymes are expressed throughout the colon and in isolated tumors.

**Figure 6:**
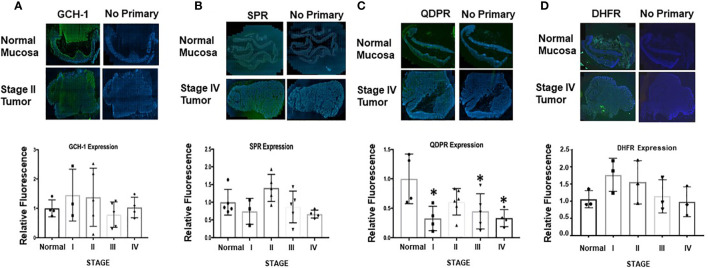
*Ex vivo* analysis of GCH-1, SPR, QDPR, and DHFR in normal mucosa and colon adenocarcinoma human tissue samples. **(A–D)** Representative sections of whole tissue sections analyzed for normal and tumor tissue by a pathologist as shown in Supporting Information **Figure 5**. In the bar graphs, GCH-1, SPR, and QDPR expression levels from five patient samples and for DHFR from three patient samples were measured for each tumor stage and normalized to DAPI. These values were subsequently compared to normal tissue expression set to 1. Relative expression is shown for each enzyme at different stages of tumor progression. Values are shown as ± SD, and unpaired t-test was used to determine statistical significance (*p<0.05).

The immunohistochemical analysis was repeated with human CRC biopsies. H&E-stained sections were delineated into regions of normal and abnormal histology by a pathologist. These histological classifications were used to assess the relative expression levels in normal and diseased areas as a function of tumor stage (**Supplementary Information Figure 3**). The patient samples were stained for GCH-1, SPR, QDPR, and DHFR. Results from representative sections of each stage of tumor progression and quantified relative expression levels for all samples as a function of tumor stage are shown in **Figure 6**. The most notable finding is the significantly decreased expression of QDPR found in stage 1 that persists throughout tumor progression. QDPR is associated with regeneration of BH4 from an intermediate, quinoid dihydrobiopterin, formed during the catalytic cycle of aromatic amino acid hydroxylases and not NOS. As a cofactor for NOS, BH4 is oxidized to a protonated trihydrobiopterin cation radical which is subsequently reduced in the next catalytic cycle by the NOS flavins. However, QDPR is ubiquitously expressed in most if not all cells and as discussed below may likely play a key role in maintaining cellular BH4 levels under conditions of oxidative stress (30, 32–35).

## Discussion

**Figure 7:**
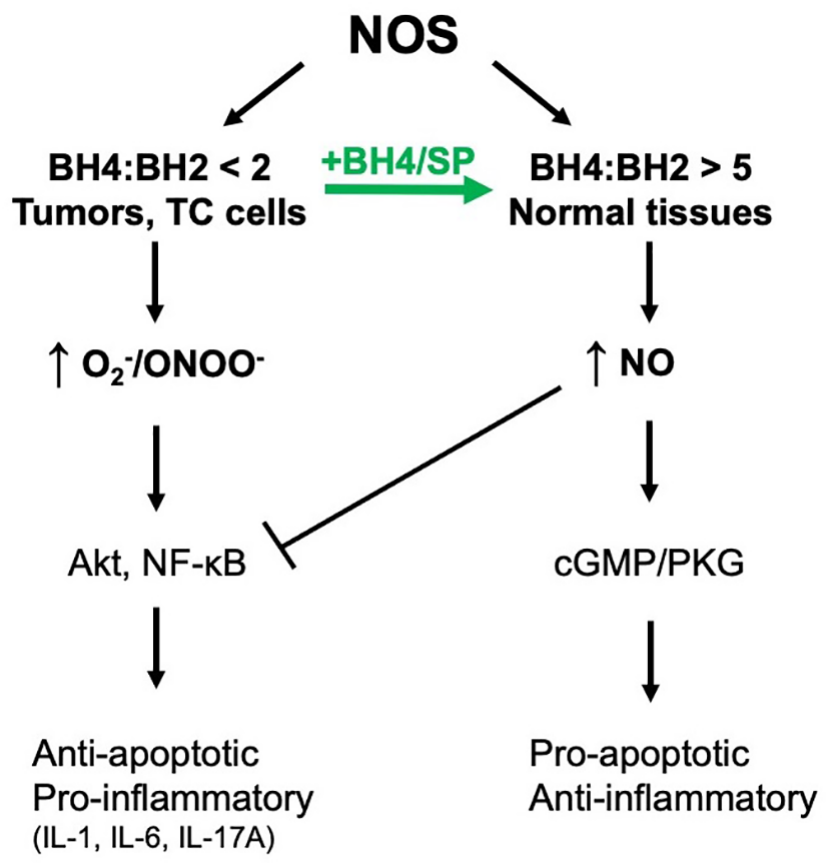
NOS coupling and NO/RNS-dependent downstream signal transduction. We have demonstrated that NOS is uncoupled in tumor cells and can be recoupled with exogenous BH4 or SP resulting in a switch from pro-survival/pro-inflammatory to anti-survival/anti-inflammatory.

Herein, it is demonstrated that treating colorectal tumor cells with SP *in vitro* and *in vivo* increases BH4:BH2 and recouples NOS activity. One consequence is the S-nitrosylation and inhibition of Akt activity and decreased phosphorylation at the inhibitory site of GSK3β. The activation of GSK3β in colorectal tumors in mice treated with SP contributes to decreased β-catenin expression and to reduced tumor growth as assessed by enhanced apoptosis and reduced [^18^F]-FDG uptake measured by PET/CT. Given the multiple mechanisms by which ROS/RNS can modulate growth promoting pathways, other mechanisms for the pro-tumor growth activity of SP are also likely involved, e.g., inhibition of NFκB signaling (17). This is evident from the experimental results in **Figure 4**, where SP induced cell death in HCT116 cells was only partially inhibited by GSK3β knockdown. **Figure 7** shows a graphical representation of our findings in this study.

Several studies including our own show that inhibiting NOS leads to a decrease in tumor growth and proliferation (26, 36–38). Others, however, have demonstrated that activating NOS or treating cells with •NO donors enhances tumor cell proliferation (39–42). One explanation for these apparently conflicting reports is the state of NOS coupling. In all solid tumors examined to date, NOS has been shown to be uncoupled producing ROS/RNS at levels that are potentially growth promoting, for example, by activating NFκB transcriptional activity (17, 43). Thus, inhibition of uncoupled NOS by decreasing ROS/RNS production inhibits activation of proliferative pathways, as seen with L-NNA, a selective NOS inhibitor (26). When *NO donors are added to cells with an uncoupled NOS generating ${\text{O}}_{2}^{-}$ , the released *NO reacts with ${\text{O}}_{2}^{-}$ producing ONOO^−^ and activating pro-proliferative and anti-apoptotic pathways, e.g., NFkB activation (43, 44).

Activation of the protein kinase G pathway of CRC and breast cancer cells with cGMP phosphodiesterase inhibitors (e.g., sulfinadic) or by overexpressing protein kinase G results in decreased β-catenin expression and inhibition of tumor cell growth (45, 46). We have previously shown with breast cancer cells that SP by recoupling NOS also enhanced cGMP/protein kinase G signaling resulting in downregulated β-catenin expression (17). In contrast, SP treatment of HCT-116 cells did not lead to an increase in cGMP levels or protein kinase G signaling in our hands (unpublished data). One explanation comes from the growing evidence that CRC cells overexpress various cGMP phosphodiesterase isoforms (13, 47). Thus, future investigations will involve combining cGMP phosphodiesterase inhibitors and SP to determine whether this will further inhibit tumor growth.

In mammary carcinoma cells, the NOS inhibitor, L-NNA, increased the BH4:BH2 ratio indicating that NOS generated ROS/RNS was, in part, responsible for the low BH4:BH2 ratio (17). The present investigation reveals an alternative mechanism in CRC cells. The expression of key biopterin metabolizing enzymes was shown to be perturbed as the tumors progressed in both mice and humans. In mice, expression of GCH1, SPR, and QDPR was shown to be significantly downregulated in AOM/DSS tumors. In humans, QDPR was also significantly downregulated. Thus, in CRC tumors, a low BH4:BH2 ratio is, in part, due to a reduced recycling of BH2 back to BH4. However, the expression levels of DHFR, which are known to reduce BH2 to BH4, remained unchanged. Recent studies with a QDPR knockout mouse provide a potential explanation (32). In the QDPR knockout mouse, a decline in the BH4:BH2+biopterin ratio was observed in all tissues except the kidney when compared to the ratio in the wild-type mouse. Furthermore, a methotrexate-induced decline in the ratio was observed in all tissues of the QDPR-deficient mouse but only in the kidney of the wild-type mouse. Given the relatively low affinity of mouse and human DHFR for BH2 and these findings with the QDPR knockout mouse, QDPR deficiency may partially account for the reduced BH4:BH2 found in tumor tissues (33). DHFR may be critical in reducing BH2 to BH4 under conditions of elevated intracellular BH2 as when cells are incubated with SP.

Future studies will need to explore alternative mechanisms of NOS uncoupling including conditions of low [Arg] as found with elevated expression of arginase or S-glutathionylation of eNOS (48, 49). Regardless of the mechanism, NOS uncoupling represents a critical switching mechanism for tumor cell growth, initiating and sustaining different downstream signaling pathways that are pro-proliferative and anti-apoptotic, e.g., NF-κB and β-catenin. When coupled, the primary product of all NOS isoforms is *NO, and downstream signaling is dominated by *NO-dependent anti-proliferative pathways (e.g., soluble guanylate cyclase/protein kinase G). In addition, we have recently shown that SP normalizes MMTVneu breast tumor vasculature increasing tumor blood flow and oxygenation and in the process enhancing tumor uptake and cytotoxicity of daunorubicin and tumor radiosensitivity (50). This shift in NOS activity thus represents an anti-tumor target that is potentially exploitable by repurposing a therapeutic already in clinical use. Synthetic BH4 (Kuvan) is used to treat a certain type of phenylketonuria and is in clinical trials for some cardiovascular diseases including patients with endothelial dysfunction. SP has also been used in phase I and II clinical trials at five times the concentrations that we are using here and was well tolerated with minimal toxicity and no reports of serious side effects.

## Data availability statement

The original contributions presented in the study are included in the article/**Supplementary Material**. Further inquiries can be directed to the corresponding author.

## Ethics statement

The animal study was reviewed and approved by IACUC.

## Author contributions

Conception and design: AA and CR. Experiments: AA, MM, VY, GS, SS, and CR. Data analysis and interpretation: AA, SS, GS, VY, and CR. Writing and review: AA, VY, and CR. All authors contributed to the article and approved the submitted version.
