# Supplementary material for: Uncoupled nitric oxide synthase activity promotes colorectal cancer progression
Source: Front Oncol. 2023 Mar 14;13:1165326. doi: 10.3389/fonc.2023.1165326 (PMC10046306; doi:10.3389/fonc.2023.1165326)
Supplement: Supplementary file 1 [file DataSheet_1.pdf]

Supplementary Information

Uncoupled nitric oxide synthase activity promotes colorectal cancer progression

Asim Alam, Steven C. Smith, Sundaresan Gobalakrishnan, Mina McGinn, Vasily A.Yakovlev, Christopher S. Rabender

Table 1 BH4:BH2 after treating cells with inhibitors of potential ROS/RNS sources.

Figure 1 Gross examination and *ex vivo* analysis of apoptosis in CAC tumors.

Figure 2 SP treatment decreases pSer^{473} Akt and increases GSK--3β activity.

Figure 3 Delineation of tumor versus normal tissue in human CRC samples

Figure 4 Immunofluorescent staining of CBR1, CBR3 and AKR1C3

Table 1: BH4:BH2 after inhibition of sources of ROS/RNS.

	BH4:BH2
**Normal Colon Tissue**	**7.1 ± 0.6**
**Control HCT116 Cells**	**2.3 ± 0.2**
**L-NNA**	**2.9 ± 0.1**
**EUK134**	**2.8 ± 0.1**
**GP91 NOX Inhibitor**	**2.7 ± 0.1**
**GP91 + EUK134 + L--NNA**	**3.1 ± 0.3**

Supplementary Table 1: BH4:BH2 after treating cells with inhibitors of potential ROS/RNS sources**.** Comparison of BH4:BH2 ratios after treatment of HCT116 cells with specific inhibitors of ROS/RNS. L--NG--nitro--L--arginine (LNNA) at 200nM for 24 hours was used to inhibit NOS. EUK134, Superoxide dismutase mimetic, was used at 10 μM for 24 hours. GP91ds--tat, NOX inhibitor, used at 5 μM for 3 hours. Values are given ± STD.

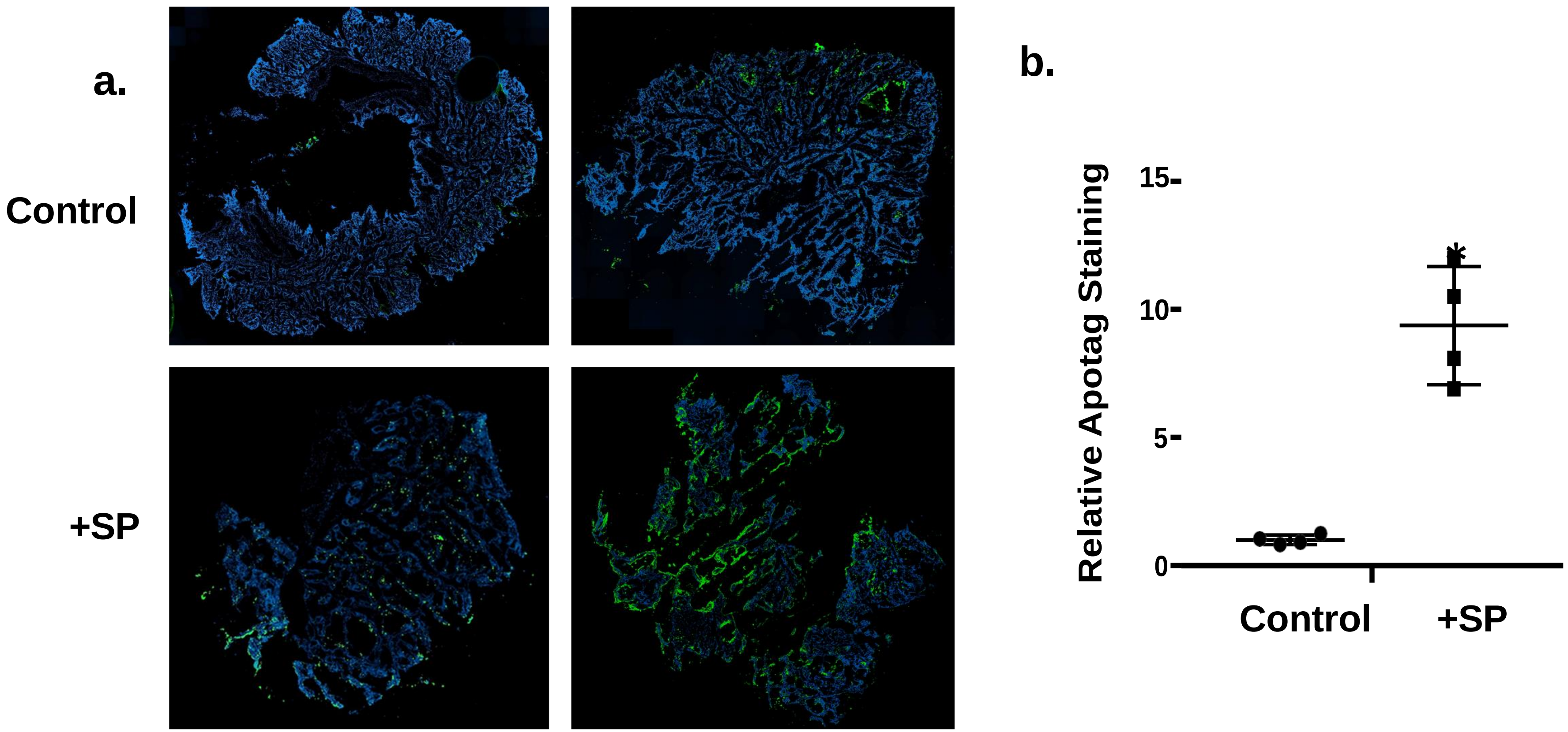

Supplementary Figure 1. Gross examination and *ex vivo* analysis of apoptosis in CAC tumors**.** At 10 weeks post AOM injection (2 weeks post final DSS treatment) SP treated animals were administered with 10mg/kg/L in drinking water with SP for 3 weeks. After treatment, animals were sacrificed and their colons removed. Tumors were excised from colon and histological slides were made and stained using ApoTAG Kit from Millipore. a) Representative sections are shown, with ApoTAG staining in green and Hoescht stain for nuclei in blue. b) Quantification was done by obtaining green:blue ratios (green ApoTAG staining to blue nuclei staining to account for size differences in tumors). Ratios were then averaged within Control (N =4) and SP treated animals (Tx) (N = 4) groups. Values are shown ± SEM; *p< 0.05

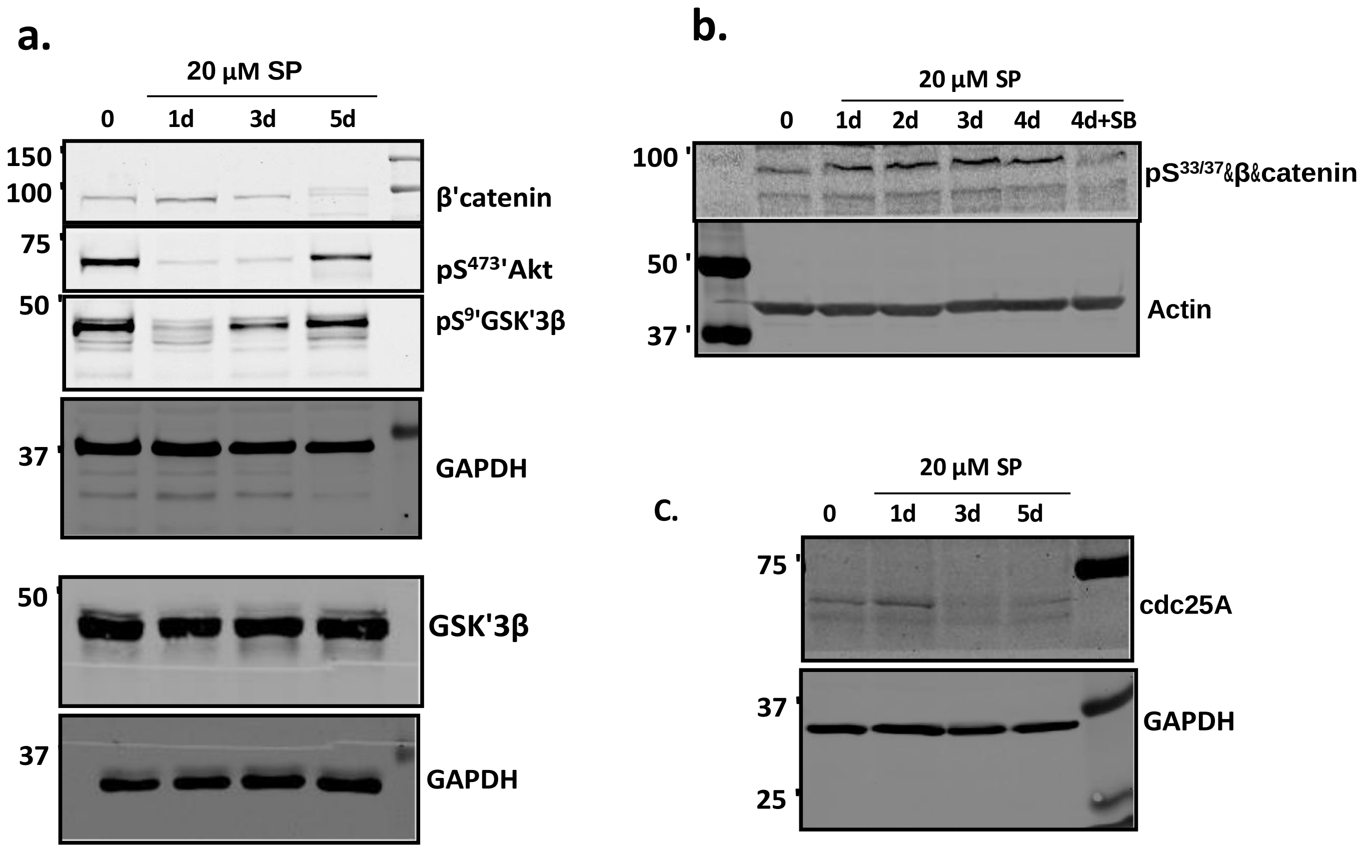

Supplementary Figure 2: SP treatment decreases pSer473--Akt and increases GSK--3β activity.
a. HT29 cells were treated with SP at 20μM for 1, 3 and 5 days. Cells were then lysed and protein extracts analyzed by western blot. Representative blots are shown from experiments done twice in this cell line. b) HCT--116 cells were treated with 20μM SP for specified days and analyzed for phosphorylation of β--catenin at Ser 33/37 (+SB = SB 216763 (GSK--3β inhibitor) plus SP for 4 days). c) cdc25A expression in HCT--116 cells. Representative blots are shown from experiments done at least 3 times in HCT--116 cells.

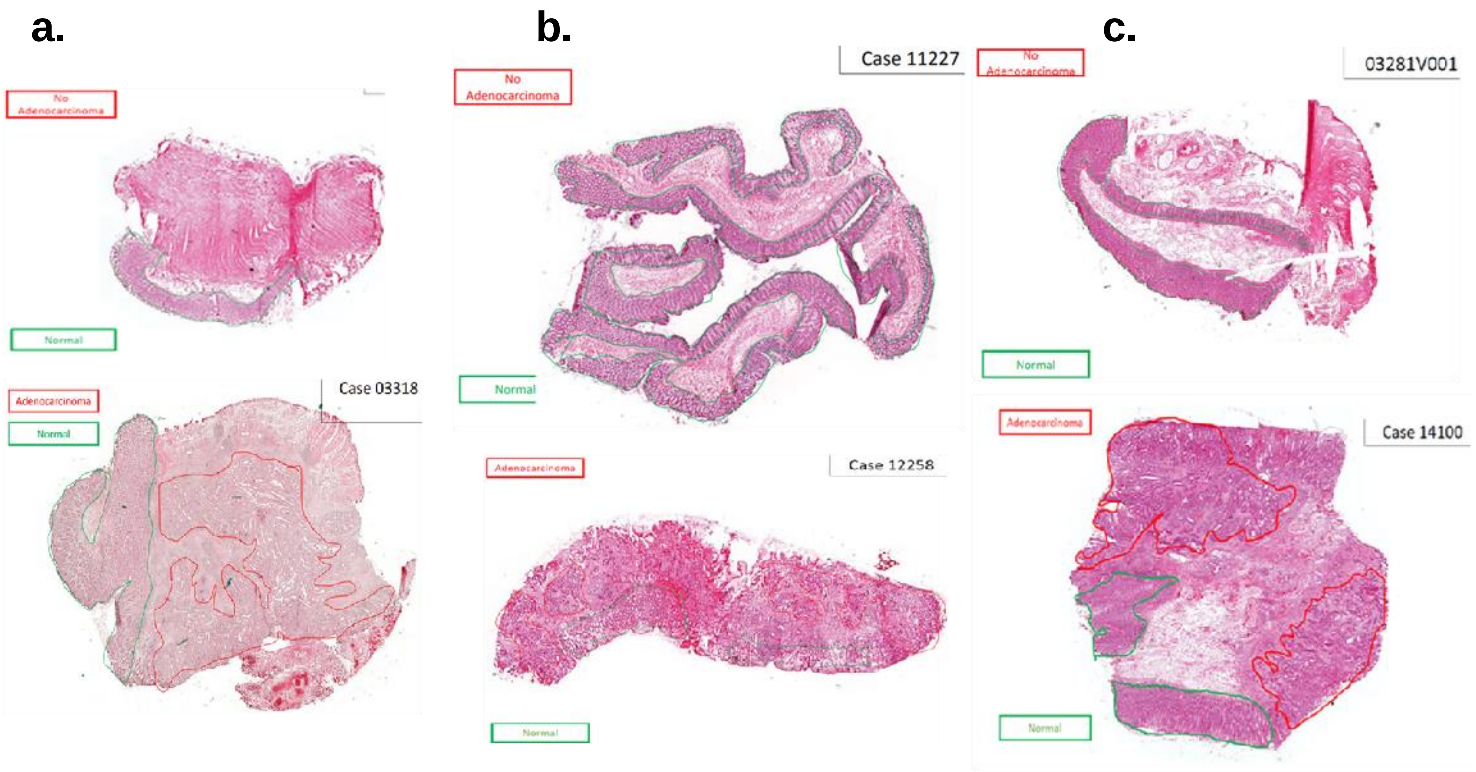

Supplementary Figure 3: Delineation of tumor versus normal tissue in human CRC samples. Five samples of normal mucosa were obtained from patients with Stage I adenocarcinoma and 5 samples each, from patients with Stage I--IV colon adenocarcinoma. H&E sections were analyzed by a pathologist for normal vs. adenocarcinoma tissue, and representative sections of this analysis are shown in this figure. Slides from columns a and b correspond to adjacent sections cut for Fig. 6A (GCH--1) and 6B (SPR), and column c for Fig. 6C (QDPR) and 6D (DFHR).

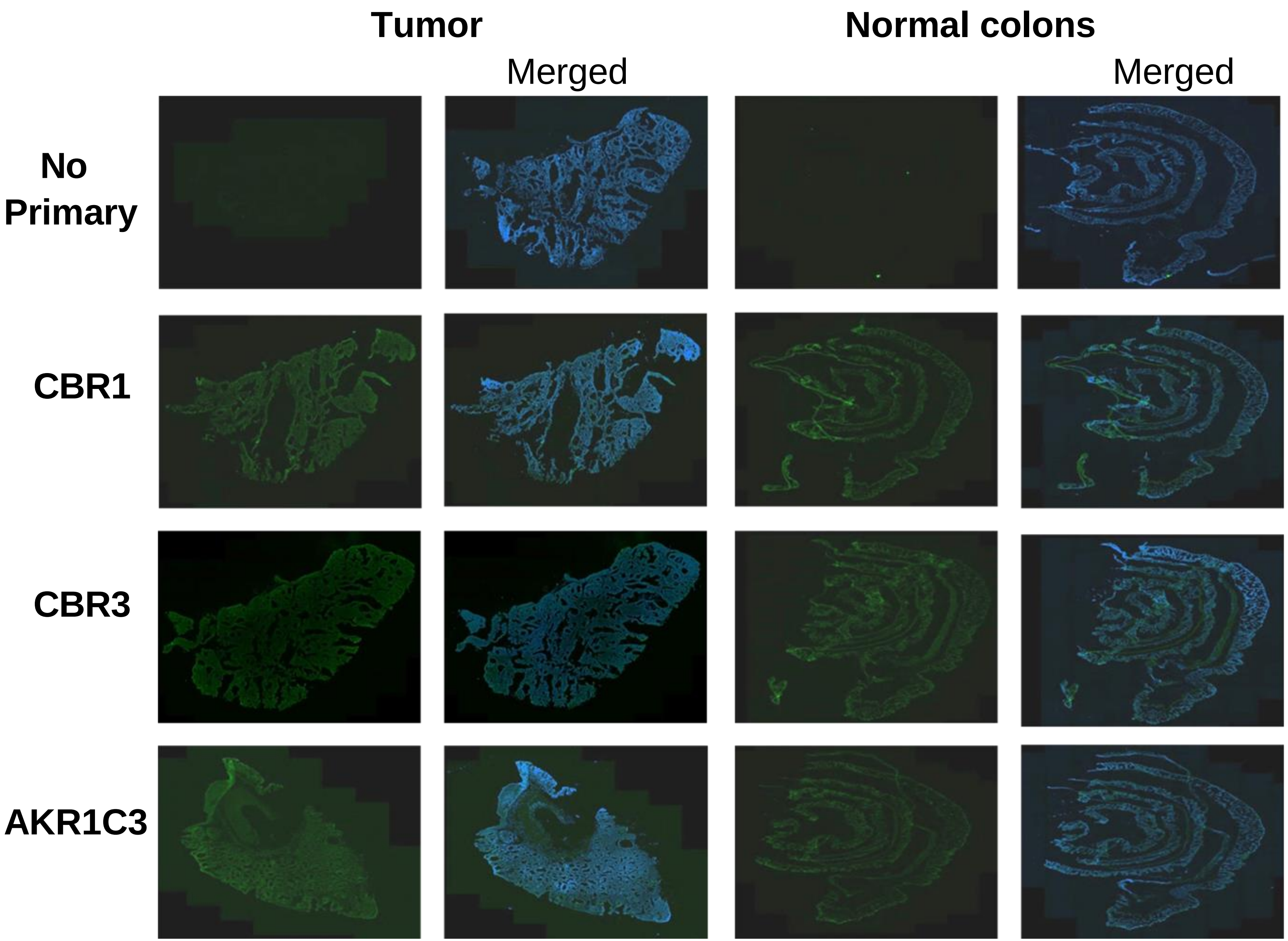

Supplementary Figure 4: Immunofluorescent staining of CBR1, CBR3 and AKR1C3. Tumors and colons from control animals were harvested and probed for CBR1, CBR3 and AKR1C3. Representative sections are shown of each molecule.
